# Muscle synergies for the control of single-limb stance with and without visual information in young individuals

**DOI:** 10.1186/s13102-021-00392-z

**Published:** 2021-12-24

**Authors:** L. Labanca, M. Ghislieri, M. Knaflitz, G. Barone, L. Bragonzoni, V. Agostini, M. G. Benedetti

**Affiliations:** 1 Physical Medicine and Rehabilitation Unit, IRCCS Istituto Ortopedico Rizzoli, Via Giulio Cesare Pupilli 1, 40136 Bologna, Italy; 2 Department of Biomedical and Neuromotor Sciences, University of Bologna, Bologna, Italy; 3 Department of Electronics and Telecommunications, Politecnico di Torino, Turin, Italy; 4 PoliToBIOMed Lab, Politecnico di Torino, Turin, Italy; 5 Department of Life Quality Studies, University of Bologna, Bologna, Italy

**Keywords:** Balance, Postural control, Postural adjustments, Muscle activations, Muscle recruitment, Postural strategies

## Abstract

**Purpose:**

Single-limb stance is a demanding postural task featuring a high number of daily living and sporting activities. Thus, it is widely used for training and rehabilitation, as well as for balance assessment. Muscle activations around single joints have been previously described, however, it is not known which are the muscle synergies used to control posture and how they change between conditions of normal and lack of visual information.

**Methods:**

Twenty-two healthy young participants were asked to perform a 30 s single-limb stance task in open-eyes and closed-eyes condition while standing on a force platform with the dominant limb. Muscle synergies were extracted from the electromyographical recordings of 13 muscles of the lower limb, hip, and back. The optimal number of synergies, together with the average recruitment level and balance control strategies were analyzed and compared between the open- and the closed-eyes condition.

**Results:**

Four major muscle synergies, two ankle-dominant synergies, one knee-dominant synergy, and one hip/back-dominant synergy were found. No differences between open- and closed-eyes conditions were found for the recruitment level, except for the hip/back synergy, which significantly decreased (p = 0.02) in the closed-eyes compared to the open-eyes condition. A significant increase (p = 0.03) of the ankle balance strategy was found in the closed-eyes compared to the open-eyes condition.

**Conclusion:**

In healthy young individuals, single-limb stance is featured by four major synergies, both in open- and closed-eyes condition. Future studies should investigate muscle synergies in participants with other age groups, as well as pathological conditions.

**Supplementary Information:**

The online version contains supplementary material available at 10.1186/s13102-021-00392-z.

## Introduction

The ability to maintain single-limb stance is essential during daily living activities and sport practice, as a single task as well as a component of other more complex tasks. It is a simple but challenging task for posture control and for this reason it is widely used for training and rehabilitation [1, 2]. In research and clinical practice, it is widely used as a testing task as it allows to quantify balance alterations and deficits of the single limb otherwise concealed during the performance of double limb tasks [3–8].

From a physiological point of view, single-limb stance can be considered as a high demanding postural task for neuromuscular and central nervous systems (CNS) requiring an efficient integration of somatosensory, visual, and vestibular information with the aim to orchestrate a continuous and effective motor response to manage a reduced base of support [9]. The effectiveness of postural control has been usually expressed by means of mechanical parameters such as the center of pressure (COP), joints or body segments displacement [10–12]. Previous literature has reported the essential role of the ankle for postural stabilization in particular when tasks show an increase in instability, as in the transition from double- to single-limb stance [13] or from stable to unstable surfaces [5]. When the ankle movements are not sufficient to guarantee balance, the involvement of more proximal joints and body segments has been reported [5, 14]. Further, an increase in the instability during stance tasks has been also reported in case of a number of pathological conditions [15–17] and in case of abnormal sensitive information [17–19]. Above all, it has been shown that vision has a key role in posture control and that the lack of visual feedback or abnormal visual feedback lead to peculiar adaptations in mechanical parameters featuring postural tasks [20, 21].

Even if mechanical parameters, such as COP or joint displacement, are useful to quantify instability during postural tasks, they do not give adequate information on motor control. Essential information for motor control assessment comes from the analysis of muscles activations, which mediates CNS control and mechanical expression of movement.

While a wide number of studies investigated multi-muscles activations during double limb stance, in the transition from double to single stance or during various stance tasks in response to sudden perturbations [22–26], less is known about quiet single-limb stance. Few studies focused on ankle/foot muscles activation, given their important role as previously described [27, 28].

However, the investigation of muscle activations around a single joint is reductive, since it is well known that CNS organizes motor response to a given task in terms of muscle synergies [29, 30]. This means that CNS coordinates the activation of a set of muscles which are synergistic for a given task, or a number of similar tasks [26].

To the best of the authors’ knowledge, it is not known which are muscle synergies used for maintaining balance condition during single-limb stance and how muscle synergies change in condition of lack of visual feedback. Since the single-limb stance task is largely used, understanding which are the muscle synergies adopted by healthy individuals is essential to address future research as well as training, rehabilitation, and functional assessment, both in healthy and pathological individuals. Thus, the first aim of this study was to investigate muscle synergies in lower limb and back muscles during a single-limb stance task without external perturbations in healthy young individuals. The second aim of this study was to investigate how the lack of visual information affects steady single-limb stance muscle synergies. Studies on the effects of visual feedback on synergistic muscle activation during double-limb stance found a change in neural drive to synergistic muscle groups with the lack of visual information [31]. It is not known how muscle synergies changes in condition of lack of visual information during single-limb-stance. Since the ankle is the first joint which acts to maintain postural stability, it is hypothesized that muscles activations around the ankle joint, and thus ankle-dominant synergies, will be affected by the greater instability related to the lack of visual information.

## Materials and methods

### Participants

Eleven male participants (age: 23.9 ± 2.2 years; height: 182 ± 8.4 cm; body mass: 74.5 ± 10.8 kg) and eleven female participants (age: 24.5 ± 2.9 years; height: 169 ± 5.8 cm; body mass: 57.2 ± 6.5 kg) were recruited to participate in the study. Inclusion criteria were: (a) age between 20 and 35 years, (b) physical activity level of 2 and 3 according to the Saltin and Grimby scale [32], thus excluding sedentary individuals and competitive athletes, and (c) absence of known neurological diseases. Exclusion criteria were (a) previous injuries or surgery, and (b) abnormalities in lower limb and foot joints.

Each participant signed an informed consent before participating in the study. The study was conducted in accordance with the Declaration of Helsinki and received ethical approval from the Ethical Committee of the Rizzoli Orthopedic Institute (PG n. 0004167).

### Experimental protocol and data analysis

**Fig. 1:**
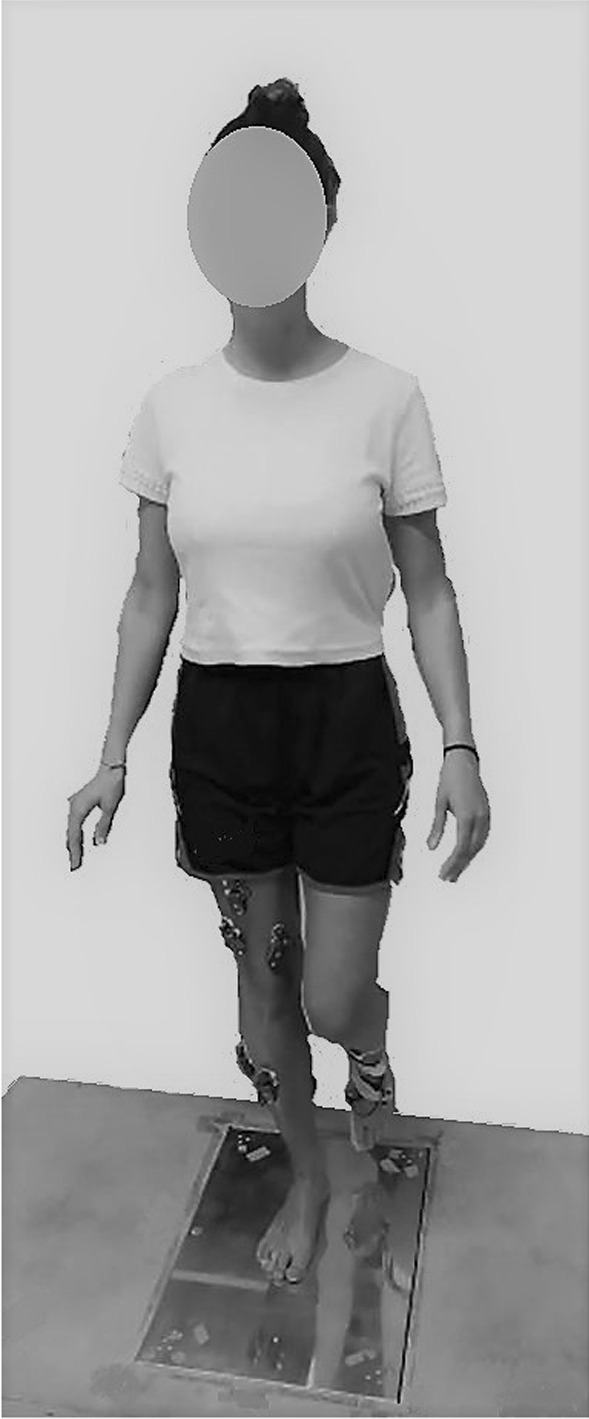
One of the participants performing the single-limb stance task

Participants were asked to stand barefoot on a force platform (Dynamic Walkway P6000, BTS Bioengineering, Milan, Italy) with the dominant limb and to maintain the contralateral knee joint flexed at approximately 90°. They were asked to look forward, to maintain upper limbs aligned to the trunk, and to remain as still as possible for at least 30 s (Fig. 1). Minimal arms movements were allowed; however, participants were asked to minimize them as much as possible. They performed the task in both opens eyes (OE) and closed eyes (CE) conditions. Two trials for each condition were performed in random order and with two minutes of rest between the trials. Muscle activations were recorded from 13 muscles of the dominant limb and trunk through electromyography wireless probes (BTS FreeEMG 1000, BTS Bioengineering, Milan, Italy) fixed on EMG electrodes (Ag/AgCl) applied over Tibialis Anterior (TA), Peroneus Longus (PL), Peroneus Brevis (PB), Soleus (SO), Lateral Gastrocnemius (LG), Vastus Medialis (VM), Vastus Lateralis (VL), Rectus Femoris (RF), Biceps Femoris (BF), Semitendinosus (ST), Gluteus Medius (GM), Longissimus Dorsii Omolateral to the dominant lower limb (LDO), and Longissimus Dorsii of Contralateral side (LDC) in accordance with SENIAM recommendations [33]. To reduce the skin impedance, before electrode application, the skin area was shaved and cleaned with ethyl alcohol. A footswitch (FSW) was placed under the first metatarsal head of the non-dominant foot. Force platform, EMG, and FSW signals were part of the same integrated system and were recorded with a 1000 Hz sampling rate.

### Segmentation of single-limb stance epochs

The segmentation of the EMG time-instants relative to the beginning and the end of the single-limb stance task was performed considering the FSW signals. More specifically, the FSW signals were used to detect the time-instants when the subject moved from the double- to the single-limb stance (beginning of the task) and vice versa (end of the task).

First, the FSW signals were amplitude-normalized to obtain signals that range between 0 and 1, where 0 corresponds to an open FSW (foot not touching the force platform) and 1 corresponds to a closed FSW (foot on the force platform). The beginning of the single-limb stance task was detected in correspondence of a 1-to-0 transition, while the end was detected in correspondence of a 0-to-1 transition. Moreover, to avoid the segmentation of excessive unipedal perturbations due to double- to single-limb stance transition (and vice versa), the beginning and the end of the single-limb stance were set 5 s after and before the previously detected time-instants, respectively.

### Muscle synergy extraction and sorting

Muscle synergy extraction and sorting procedures were performed in accordance with our previous study [34]. Briefly, the segmented EMG signals corresponding to single-limb stance tasks were high-pass filtered at a cut-off frequency of 35 Hz through an 8th order zero-lag IIR Butterworth digital filter, full-wave rectified, and low-pass filtered at a cut-off frequency of 12 Hz through a 5th order zero-lag IIR Butterworth digital filter [35]. The EMG amplitude was normalized to the global maximum activation of each muscle recorded for each trial of each condition to ensure the equally weighted contribution of all the observed muscles in the muscle synergy assessment [35].

The original data matrix containing the envelopes of the segmented EMG signals was then factorized into low-dimensional elements using the Non-negative Matrix Factorization (NMF) algorithm [30, 36]. The NMF models the original data matrix as the linear combination of two low-dimensional elements [37]: the time-independent weight vectors (*W*) modeling the spatial component of the motor control and the time-dependent activation coefficients (*C*(*t*)) modeling the temporal component of the motor control, as detailed in (1):

1 $$M(t) = \sum\limits_{k = 1}^{N} {C(t)_{k} \cdot W_{k} + e}$$

where *N* represents the number of muscle synergies needed to accurately assess the motor control and $e$ is the reconstruction error.

The reconstruction accuracy of the original EMG signals for each number of synergies from 1 to 8 was computed through the total Variance Accounted For (*tVAF*), defined as the uncentered Pearson’s correlation coefficient. The *tVAF* was used to select the optimal number of muscle synergies (*N*_*opt*_) needed to properly reconstruct the original EMG signals and to accurately assess the motor control strategies. As detailed in our previous work [34], the *N*_*opt*_ was selected by consecutively applying a global criterion on each number of synergies from 1 to 8 (least number of synergies granting a *tVAF* ≥ 90%) and a local criterion on the number of muscle synergies selected through the global criterion (*VAF* ≥ 75% for each of the observed muscles) [30, 38, 39].

Muscle synergies extracted from each trial of each condition were then sorted through a *k*-means clustering algorithm applied to the weight vectors (*W*) by setting the *k* value equals to *N*_*opt*_ [40]. Once the weight vectors were sorted, the activation coefficient vectors (*C(t*)) were ordered consequently.

### Muscle synergy analysis

Muscle synergies extracted from the segmented EMG signals during the two different task conditions (OE and CE) were quantitatively compared in terms of (i) the optimal number of muscle synergies (*N*_*opt*_), (ii) the average recruitment levels (*Recr*), and (iii) balance control strategies (*S*).

i. Optimal number of muscle synergies (*N*_*opt*_)

The optimal number of muscle synergies (*N*_*opt*_) was selected for each trial of each task condition by choosing the smallest number of muscle synergies (*N*) which guarantees t*VAF* ≥ 90% (global criterion) and *VAF* ≥ 75% (local criterion) for each of the acquired muscle.

ii. Average recruitment levels (*Recr*)

Since no typical cyclostationary processes can be assessed during a single-limb stance task, the activation coefficients (*C(t*)) were compared in terms of average recruitment level (*Recr*_*k*_), defined as the average (over time) of each activation coefficient vector *C*(*t*)_k_ [30, 41]. The recruitment level values range between 0 (no recruitment) and 1 (maximum recruitment) and quantify how much a specific muscle synergy is activated in the execution of the task.

iii. Balance control strategies (S)

Three different balance control strategies were defined considering the acquired muscles: ankle control, knee control, and hip/trunk control strategy. The ankle control strategy (*S*_*ankle*_) was mainly identified by the activation of 5 leg muscles (PL, PB, LG, TA, and SO), the knee control strategy (*S*_*knee*_) by the activation of 3 shank muscles (VM, VL, and RF), and the hip/trunk control strategy (${S}_{hip}$) by the activation of 5 muscles of the proximal lower limb and the trunk (BF, ST, GM, LDO, and LDC). The computation of the balance control strategies is described in detail in our previous study [34].

### Statistical analysis

To assess statistically significant changes in the optimal number of muscle synergies considering the two different task conditions (OE and CE), the hypothesis of normality of the distribution was first tested through the Lilliefors test. If the normality hypothesis was rejected, the Wilcoxon signed-rank test was performed, otherwise, a two-tailed paired Student’s *t*-test was performed. Two-way ANOVA for repeated measures followed by *post-hoc* analysis with Tukey adjustment for multiple comparisons was performed to evaluate the differences between conditions (OE and CE) and muscle synergies (factors: condition and synergies), for both the average recruitment levels (*Recr*) and balance control strategies (*S*). For the weight vectors (*W*), an analogous two-way ANOVA was applied to evaluate the differences between conditions and muscles. All the levels of significance (α) were set equal to 0.05. The statistical analysis was carried out using the Statistical and Machine Learning Toolbox of MATLAB^®^ release R2020b (The MathWorks Inc., Natick, MA, USA).

## Results

As follows, are reported the muscle synergy results computed considering the two different single-limb stance conditions (OE and CE). An example of the activation coefficients and weight vectors obtained from one of the participants in the eyes open and eyes closed conditions has been reported as additional data (see Additional file 1). More specifically, muscle synergies were compared in terms of (i) the optimal number of muscle synergies, (ii) average recruitment levels, and (iii) balance control strategies.

i. Optimal number of muscle synergies (N_opt_)

The application of the Wilcoxon signed-rank test revealed no statistically significant differences (p = 0.52) in the optimal number of muscle synergies (*Nopt*) between the OE and CE conditions. In particular, 4 muscle synergies were needed to accurately model the motor control strategies during both the OE and CE conditions.

**Fig. 2:**
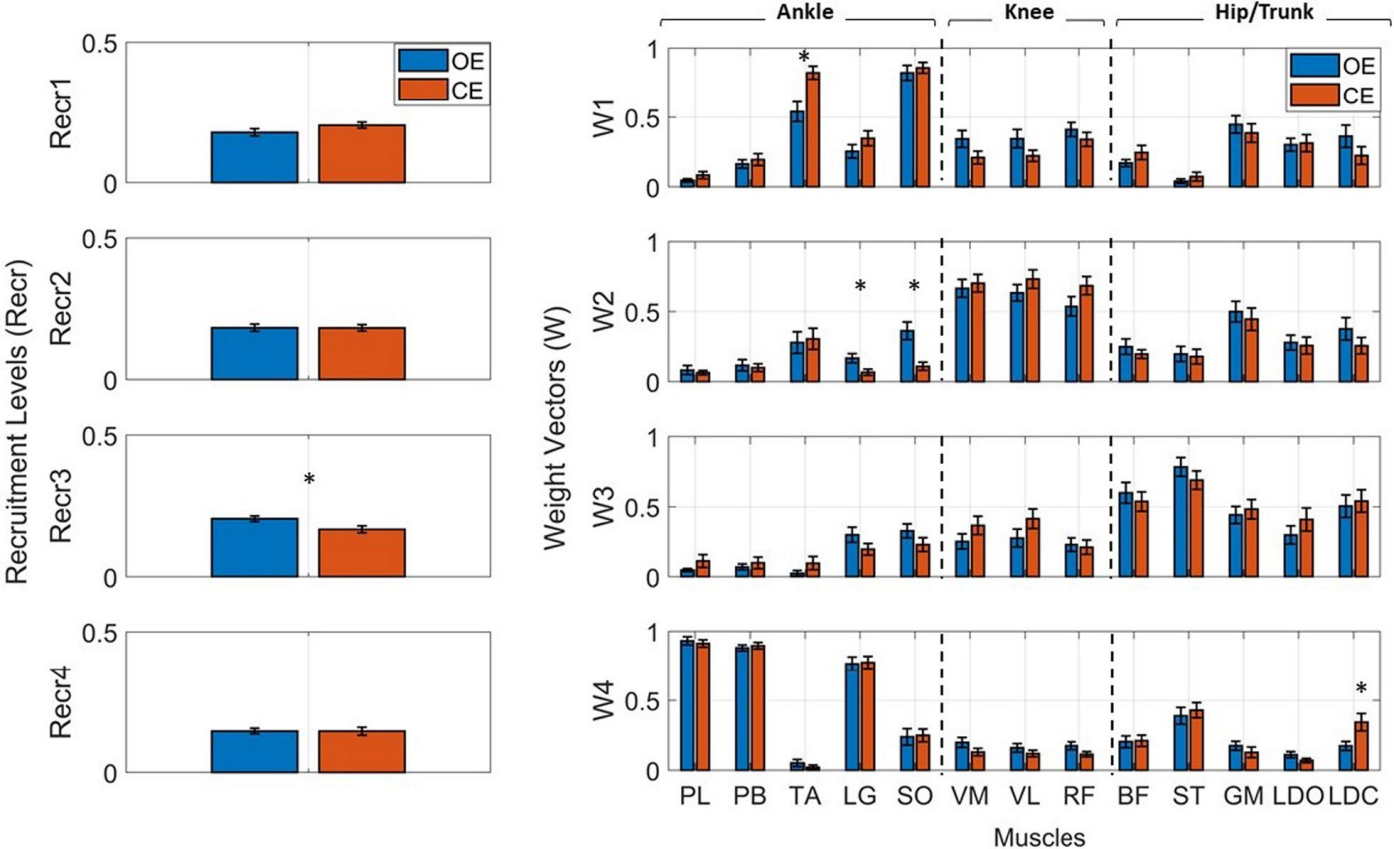
Comparison of muscle synergies extracted during eyes open (OE) and eyes closed (CE) single-limb stance conditions. Color vertical bars represent average recruitment levels *Recr*_*k*_ (on the left) and weight vectors *W*_*k*_ (on the right) of the *k*-synergy, over the sample population, with the superimposition of the standard errors (black lines). The asterisk (*) represents a statistically significant differences between conditions, in the weight vectors and average recruitment levels

Figure 2 shows the muscle synergies, averaged over the sample population, extracted from the two different task conditions: OE represented in blue and CE in red. More specifically, for each muscle synergy, the average recruitment levels *Recr*_*k*_ (on the left) and the weight vectors *W*_*k*_ (on the right) are represented. The asterisk (*) indicates statistically significant differences between conditions (repeated measures two-way ANOVA, *p* < 0.05), both for the average recruitment levels and weight vectors.

ii. Average recruitment levels (*Recr*)

A statistically significant decrease (*p* = 0.02) was found in the average recruitment level of the third muscle synergy S_*hip*_ extracted during the CE condition (0.17 ± 0.01) with respect to the OE condition (0.21 ± 0.01). No statistically significant differences were detected considering the remaining three muscle synergies between OE and CE conditions, suggesting no changes in the recruitment levels of those synergies due to the loss of visual feedback.

Figure 2 shows the average recruitment levels (on the left), over the sample population, extracted during OE and CE single-limb stance conditions.

iii. Balance control strategies (*S*)

As shown in Fig. 2, the first and the fourth muscle synergies can be mainly associated to an ankle control strategy, since the muscles mainly enrolled are those belonging to the leg (PL, PB, LGS, TA, and SO), the second muscle synergy to a knee control strategy and the third muscle synergy to a hip/trunk control strategy. Two-way ANOVA for repeated measures revealed a statistically significant increase (*p* = 0.03) of the ankle control strategies (*S*_*ankle*_) during the CE condition (0.52 ± 0.06) with respect to the OE condition (0.47 ± 0.06). No additional statistically significant differences were detected considering the remaining two balance control strategies (*S*_*knee*_ and *S*_*hip*_) between conditions.

Table 1 represents the values of the balance control strategies, averaged over the sample population, with the indication of the statistically significant changes between OE and CE conditions.

**Table 1:** Balance control strategies (*S*) averaged on the sample population

Balance control strategies	Average balance control strategies (mean ± standard deviation)		
	OE	CE	ANOVA (p-value)
*S*_*ankle*_	0.47 ± 0.06	0.52 ± 0.06	0.03
*S*_*knee*_	0.61 ± 0.24	0.71 ± 0.25	0.22
*S*_*hip*_	0.53 ± 0.15	0.53 ± 0.17	0.89

*S*_*ankle*_: ankle control strategy, *S*_*knee*_: knee control strategy, and *S*_*hip*_: hip/trunk contorl strategy

## Discussion

The results of this study show that four major muscle synergies are used during single-limb stance, i.e., two ankle-dominant synergies, one knee-dominant synergy, and one hip/back-dominant synergy, in an open-eyes as well as in closed-eyes condition. In addition, there is no difference in the recruitment level between the open-eyes and closed-eyes conditions, except for the hip/back synergy, which showed a decreased activation in the closed-eyes compared to the open-eyes condition. At the same time an increase in the ankle balance strategy was found in the closed-eyes compared to the open-eyes condition, confirming the initial hypothesis of this study.

Since the work by Horak and Nashner [42], it is widely recognized the essential role of the ankle for the control of upright posture and for the maintenance of posture when balance is challenged by perturbations of the supporting surface. In these circumstances, muscles around the ankle joint provide the first activation strategy for balance maintenance [14]. In our study, no perturbations were applied to the supporting surface and participants were required to maintain a quiet stance. The key role of the ankle for the control of posture in quiet stance is confirmed by the observation of two ankle-dominant synergies adopted by the participants in this study. The first ankle-dominant synergy (W1) is mainly featured by the tibialis anterior and the soleus muscle activation. The second ankle dominant synergy (W4) is mainly featured by peroneus longus and brevis muscles and gastrocnemius lateralis muscle activations. The two synergies may reflect the activations related to anterior posterior sway and medio-lateral sway, respectively, which may occur during a single-limb stance task. In particular, the co-activation of antagonist muscles, in this case tibialis anterior and soleus, might represent a strategy to cope with reduced base of support, with the aim to reduce movement variability and maintaining stability. Previous studies found an increase in tibialis anterior and soleus muscles activation in particular in older adults to compensate for reduced vision [43] or decreased tendon stiffness [44], and both in children and elderly which showed a diminished postural steadiness when compared with young adults [28].

Literature reports that as difficult the task becomes as higher is the involvement of more proximal joints for the maintenance of balance, in particular the hip [5, 14]. In experimental settings, the difficulty of the task is usually increased by increasing the magnitude of a perturbation, by decreasing the magnitude of the supporting surface or by changing the features of the supporting surface [5, 23, 24, 26]. For example, it has been reported that by moving from a stable to an unstable surface, the angular displacement of the ankle was stable across all the testing condition, with the knee and hip displacement arising when the difficulty of the task was higher [5, 45].

In our study, the difficulty of the task was not modified throughout the experiment and the support surface was not unstable. However, standing on a single limb might be considered as a per se difficult task because of the reduced base of support in comparison to the common double-limbs stance. Usually, when the support base is reduced, a precaution strategy consisting in moving forward the center of mass is adopted to avoid falling backward. This explains the presence of the hip/back muscle synergy (mainly featured by hamstrings and back muscles) adopted by the participants of our study. It should be also mentioned that, in a condition of quiet stance, the co-existence of the hip strategy with the ankle strategy has been reported [45], highlighting that the two strategies are not different entities, but one predominates depending upon the task and conditions of the environment [45]. It is reasonable to think that the participants of the present study used the hip/back synergy to compensate for ankle dorsiflexion used to move forward the center of mass to manage the reduced base of support.

The essential role of quadriceps muscle for balance control during single-limb stance tasks is highlighted by the presence of the knee-dominant synergy (W2) used by the participants in this study. In fact, the knee-dominant synergy was probably used when the ankle synergy was not effective for the maintenance of balance, but the condition did not require yet the involvement of the hip or the back synergy. These results highlight the fine coordination between ankle muscles and quadriceps muscle. It was observed that when the knee-dominant synergy was used, ankle muscles had in general a low activation. This was especially observed in the closed-eyes condition, when the lack of visual information led to an increase in the difficulty of the task. In fact, it was observed a significantly lower activation of the soleus and gastrocnemius muscles when the knee-dominant synergy was used. This observation arises two possible speculations. The first is that the knee synergy is used when the ankle synergy is not sufficient for balance control. The second is that knee-synergy may be effective alone to guarantee stability during single-limb stance in some circumstances. At the same time when ankle-dominant synergies are used, a low activation of the quadriceps is observed in particular when the ankle synergy is featured by evertor muscles activation. This could be explained by the fact that this synergy is mainly used to manage with medio-lateral displacement. This observation is further confirmed by the higher activation of back muscles of the contra-lateral side for back stabilization in the mediolateral direction.

However, despite some differences in the closed- compared to the open-eyes condition, the number of synergies used is the same between the conditions, as well as the level of recruitment. This is in accordance with previous literature reporting the stability of muscle synergies adopted between tasks with the variation of the visual feedback [24, 46]. It has been shown that in general the lack or the disturbance of vision does not affect synergies because during standing postural control mostly relies on proprioceptive feedback [24, 46]. In fact, the results of previous investigations show that proprioceptive disturbance, but not visual disturbances, affected the regulation of muscle synergies [24] and the increase in body sway [46].

The reduction in the recruitment level of the hip/back synergy in closed- compared to the open-eyes condition seems not in accordance with previous literature, reporting a major involvement of proximal joints as the difficulty of the task increases [5, 42, 45]. However, in the present study, an increase in the involvement of the ankle-dominant synergy for balance control has been observed in the closed-eyes condition. This result confirms the initial hypothesis of this study on the increase in muscles activations around the ankle joint. It is likely to think that this modulation aimed at decreasing the degrees of movement to increase stability, was probably sufficient to maintain balance and the use of muscle synergies involving proximal joints and segments was not determinant for the outcome of the task.

The observation of a change in the modulation of some of the muscle activations in the closed-eyes condition is in accordance with previous literature. A decrease in synergistic muscle coherence was observed during double-limb stance in a closed-eyes compared to an open-eyes condition [31], thus showing that the lack of visual feedback and the reliance on other sources of afferent information affects the generation of neural inputs on synergistic muscles. Regarding the results of the present study, it can be thus speculated that the lack of visual information affects the modulation of muscle activation, without altering the type and numbers of synergies adopted. For example, an increase in the ankle balance strategy was found in the closed-eyes compared to the open-eyes condition. It is plausible to think that the lack of the visual feedback led to a sensory reweighting for the control of posture, shifting the sensory information arising from vision with an increased proprioceptive information arising from the ankle joint and ankle movements [47].

Accordingly, the results of this study suggest also that muscle synergies are probably not exclusively managed throughout a feedforward control, but can be modulated with a feedback control based on the signals arising from sensory receptors, with the aim to correct movement errors which may occur in some circumstances. It is likely to think that the maintenance of the single-limb stance in this study was controlled with pre-programmed muscle synergies. However, the difficulty of the task leading to continuous losses and recovery of balance probably needs a continuous movement correction based on a feedback control relying on information arising from sensory receptors. Animal studies have reported organized patterns of muscles activations in response to focal stimulation of the spinal cord [48–51], thus suggesting that a feedback control may be launched at spinal level in response to specific sensory stimuli to modulate the centrally organized synergy recruitment. It is likely to think that similar patterns may regulate muscle synergies also in humans.

Finally, it should be mentioned that in this study biceps femoris and semitendinosus, which are two-joints muscles, were grouped in the hip/back synergy, and not into the knee synergy. This is related to the fact that as for the nature of the task, hamstrings muscles were more deputed to the hip extension than to knee flexion [52, 53]. At the same time, quadriceps muscle, which is also a two-joints muscle, was grouped only in the knee synergy. This is related to the fact that participants were asked to stand in an upright posture with the hip joint in full extension. In the latter position, the quadriceps (and in particular the RF) has a reduced activation and thus a lower involvement in the hip joint control [54].

To the best of the authors’ knowledge, this is the first study investigating muscle synergies deputed to the maintenance of posture during a single-limb stance task without external perturbations, in an open-eyes and closed-eyes condition. Due to the large use of this kind of task in clinical practice, both for rehabilitation and functional assessment, as well as in sport practice for training and testing, the results of the present study give important information on motor control of this kind of task in healthy individuals. Future studies should investigate muscle synergies also in other populations to investigate the effects of orthopedic and neurologic pathologies on muscle synergies, as well as the effect of rehabilitation and training.

The main limitation of this study is that we recruited only healthy young individuals, and thus the results cannot be generalized to all healthy individuals. Future studies should identify muscle synergies used for single-limb stance also in other age groups. A second limitation of the study was that muscle synergies for the transition between double- and single-limb stance (and vice versa) were not analyzed, thus the results of the present study have to be considered exclusively for steady single-limb stance tasks.

## Conclusions

In conclusion, single-limb stance is featured by four major muscle synergies, two ankle-dominant, one knee-dominant and one hip/back-dominant. The lack of visual feedback did not affect the number of synergies used. In general, an increase of activation of the ankle muscles and a decrease in the recruitment of the hip/back synergy was observed in the absence of visual information in comparison to the normal vision condition. To the best of the authors knowledge, this is the first study providing information on muscle synergies adopted during single-limb stance which is a task featuring a number of daily living activities, as well as training and rehabilitation exercises. Future studies should investigate muscle synergies during single-limb stance also in other age groups, and it seems of high clinical relevance to investigate synergies on orthopedic and neurologic patients to address clinical practice and rehabilitation interventions.

## Supplementary Information

**Additional file 1.** Example of muscle synergies in one of the participants. Activation coefficients and weight vectors obtained from a representative healthy subject of the sample population considering two different task conditions: eyes open and eyes closed conditions

## Data Availability

All data and material are available from the corresponding author upon reasonable request.

## Abbreviations

BF: Biceps femoris
C: Activation coefficients
CE: Closed eyes
CNS: Central nervous system
COP: Center of pressure
EMG: Electromyography
FSW: Footswitch
GM: Gluteus medius
LDC: Longissimus dorsii of contralateral side
LDO: Longissimus dorsii omolateral to the dominant lower limb
LG: Lateral gastrocnemius
M: Models of the original EMG envelopes
N: Number of synergies
NMF: Non-negative matrix factorization
OE: Open eyes
PB: Peroneus brevis
PL: Peroneus longus
Recr: Average recruitment level
RF: Rectus femoris
S: Balance control strategies
SO: Soleus
ST: Semitendinosus
TA: Tibialis anterior
VAF: Variance accounted for
VL: Vastus lateralis
VM: Vastus medialis
W: Weight vectors
