# Supplementary material for: Muscle synergies for the control of single-limb stance with and without visual information in young individuals
Source: BMC Sports Sci Med Rehabil. 2021 Dec 24;13:163. doi: 10.1186/s13102-021-00392-z (PMC8710023; doi:10.1186/s13102-021-00392-z)

**Additional file 1**

**Example of muscle synergies in one of the participants.**

Activation coefficients (C) and weight vectors (W) obtained from a representative healthy subject of the sample population considering two different task conditions: (A) eyes open and (B) eyes closed conditions. Activation coefficients are represented as temporal signals, time-normalized with respect to the duration of the Single Leg Stance. Weight vectors are represented trough bar diagrams. Muscle abbreviations: PL = Peroneus Longus, PB = Peroneus Brevis, TA = Tibialis Anterior, Lateral Gastrocnemius = LG, Soleus = SO, Vastus Medialis = VM, Vastus Lateralis = VL, Rectus Femoris = RF, Biceps Femoris = BF, Semitendinosus = ST, Gluteus Medius = GM, Longissimus Dorsii Omolateral to the dominant lower limb = LDO, and Longissimus Dorsii of Contralateral side = LDC.


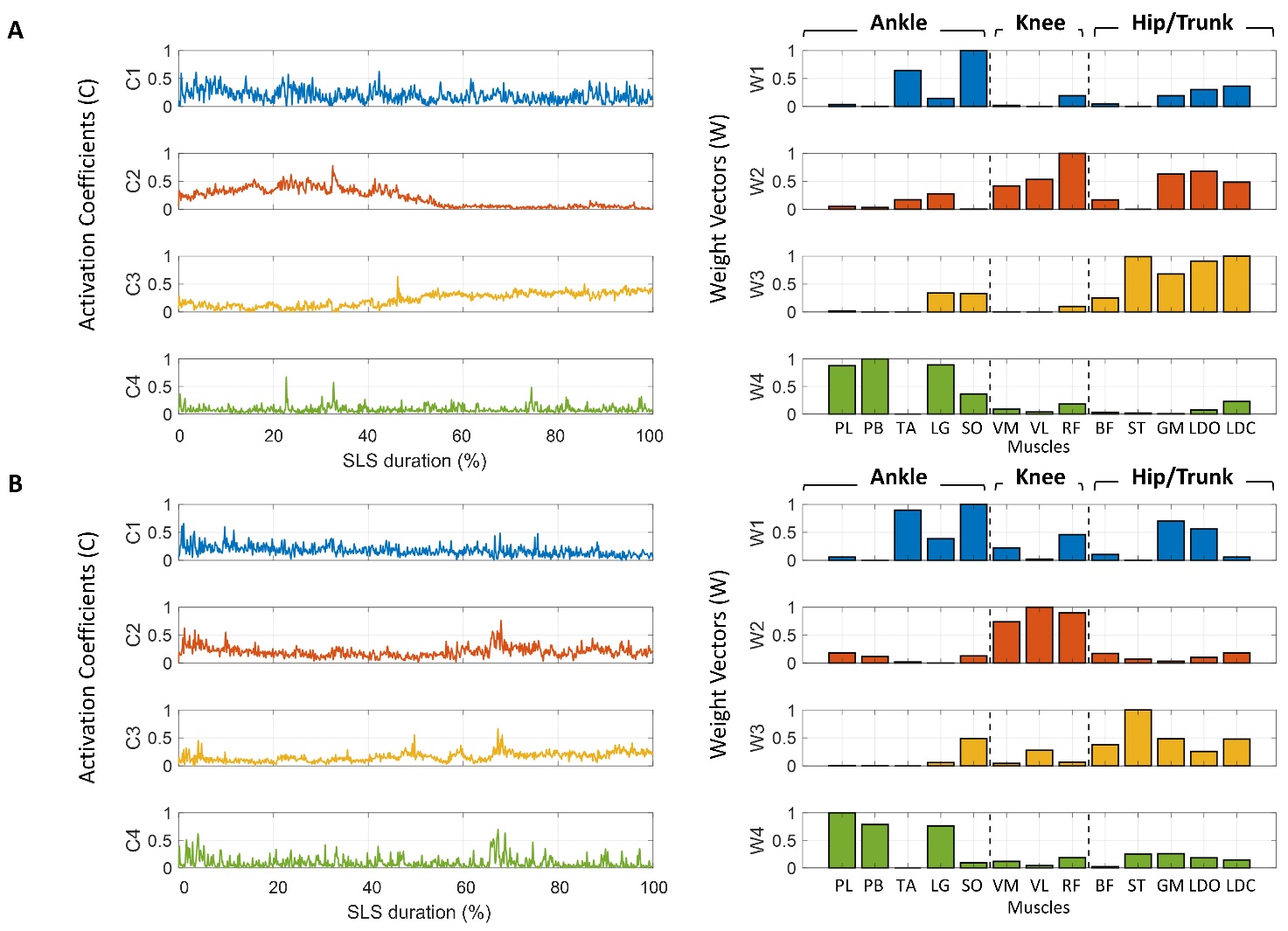

Supplement: Supplementary file 1 — Additional file 1. Example of muscle synergies in one of the participants. Activation coefficients and weight vectors obtained from a representative healthy subject of the sample population considering two different task conditions: eyes open and eyes closed conditions [file 13102_2021_392_MOESM1_ESM.docx]
